# A case of chaos? NO causes arrhythmic motor pattern via interstitial cells of Cajal in the murine colon

**DOI:** 10.1186/2050-6511-16-S1-A36

**Published:** 2015-09-02

**Authors:** Katharina Beck, Dieter Groneberg, Andreas Friebe, Barbara Lies

**Affiliations:** 1 Physiologisches Institut, Universität Würzburg, Würzburg, Germany

## Background

Gastrointestinal (GI) motility originates from the complex coordination of smooth muscle contraction and relaxation. GI diseases affecting motility are often associated with impaired nitrergic signaling. In the enteric nervous systems, NO is released from nitrergic neurons as a major inhibitory neurotransmitter. NO acts via NO-sensitive guanylyl cyclase (NO-GC) in different GI cell types such as smooth muscle cells (SMC) and interstitial cells of Cajal (ICC). The precise mechanism of nitrergic signaling through these two cell types to regulate colonic spontaneous contractions is not fully understood yet.

## Methods

Longitudinal smooth muscle of the proximal colon from WT mice exhibits spontaneous contractile activity in vitro. Colon from global and ICC-specific GCKO animals also exhibited spontaneous rhythmic contractions. Yet, in both genotypes, duration and amplitude of the rhythmic contractions were increased compared to WT. In line with that, colon from WT revealed an arrhythmic contractile pattern which was transformed into a uniform motor pattern after addition of L-NAME or ODQ. Electrical field stimulation induced off-contractions in WT and high amplitude on-contractions in global GCKO colon.

## Results and conclusion

Our results prove that basal NO release participates in the regulation of spontaneous contractions in the murine proximal colon. NO-GC activity in ICC converts the contraction pattern from periodic into irregular. Thus, NO-GC in ICC is the major effector for NO in the proximal colon.

